# Do Demographic Characteristics Make Differences? Demographic Characteristics as Moderators in the Associations between Only Child Status and Cognitive/Non-cognitive Outcomes in China

**DOI:** 10.3389/fpsyg.2017.00423

**Published:** 2017-04-04

**Authors:** Ning Liu, Yiting Chen, Xiangdong Yang, Yi Hu

**Affiliations:** ^1^Institute of Curriculum and Instruction, Faculty of Education, East China Normal UniversityShanghai, China; ^2^School of Psychology and Cognitive Science, East China Normal UniversityShanghai, China; ^3^School of Education and Psychology, Hainan Normal UniversityHaikou, China

**Keywords:** only children, only child status, cognitive outcomes, non-cognitive outcomes, demographic characteristics

## Abstract

Different family compositions and sizes may affect child development through the different modes of interaction between family members. Previous studies have compared only children with non-only children in cognitive/non-cognitive outcomes. However, relatively little research has systematically investigated the potential moderators among them. Using a large and representative sample of Chinese students (Grades 7–8; *N* = 5,752), this study examines the roles of demographic characteristics, such as gender, region, parental educational level, parental expectations, family socio-economic status and family structure, in the associations between only child status and cognitive/non-cognitive outcomes. For the cognitive outcomes, only child status exerts an influence on the students' academic performance in Chinese and mathematics in the sample of three districts' students. The examined associations between only child status and cognitive outcomes are different in region, parental education, parental expectations and family structure, while gender and family socio-economic status did not. For the non-cognitive outcomes, only child status exerts an influence on the students' school well-being, academic self-efficacy, academic self-concept, and internal academic motivation in the full sample of students, but not on external academic motivation. Further, the examined associations between only child status and non-cognitive outcomes are different in region, parental education, family socio-economic status and family structure, while gender and parental expectations did not. These findings suggest that the associations between only child status and cognitive/non-cognitive outcomes are heterogeneous in terms of some of the demographic characteristics. Possible explanations are proposed in some concepts of region and family environment in China.

## Introduction

Since the implementation of China's new family planning policy in 2016, which permits all couples to have two children, the one-child policy, which restricted urban Han Chinese couples to one child each, has ended (Peng, 1991; White and White, 2012). It had been in place for more than 30 years. During this period, China has undergone significant changes in family structure. In particular, there are many only children in the younger generation. According to the basic data from the sixth census in 2010, the number of only children reached ~164 million (Gu, 2016) and this number is currently still increasing. These significant changes in family structure prompt questions regarding the consequences of growing up without siblings and what the potential moderators might be.

Different family compositions and sizes may affect child development through the different modes of interaction between family members. It is generally believed that the family environment inevitably makes only-child groups different from non-only-child groups in terms of the children's cognition, personality, and affect characteristics (Feng, 1992; Hao and Feng, 2002; Li et al., 2013). However, relatively few studies have explored the associations between only child status and non-cognitive outcomes (academic self-efficacy, academic self-concept, academic motivation); especially the potential moderators in the associations. In addition, extant studies have primarily used data on specific subpopulations. This study investigated the associations between only child status and cognitive/non-cognitive outcomes and the possible moderators using a large, representative dataset.

### Theoretical pathways of influence

Only-child status may affect child development directly through their interaction with parents or indirectly through the siblings. Specifically, only children (as well as the first born) got the greater levels of parental responsiveness, attention, and concern, compared with non-only children (Schachter, 1959). These made them feel more security which facilitates children's development of intellectual competence, psychological confidence, and mature behavioral patterns (Ainsworth, 1982; Armsden and Greenberg, 1987). In addition, without competing with siblings, only children, as the sole recipient of family assets, were advantaged in their educational opportunities, physical health, and their general well-being (Falbo and Polit, 1986; Falbo, 1987). For example, an accumulating body of evidence have revealed that only children exhibited better academic skills (Bao and Su, 1989; Falbo and Poston, 1993; Ji et al., 1993; Falbo, 2012), had higher academic self-concept (Hu, 2009), subjective well-being and life satisfaction (Hu, 2007; Kwan and Ip, 2009; Shao et al., 2013).

Although there are many reasons to believe that only children may be not only associated with higher cognitive outcomes also with higher non-cognitive outcomes, the absence of siblings may indirectly hinder their development on cognitive/non-cognitive outcomes. For example, only-children usually missed out on important opportunities to rehearse some of the more complicated aspects of relationships within a safe environment and also missed many opportunities to develop psychosocial skills, emotional support, and learning opportunities compared with non-only-children (Dunn and Slomkowski, 1992; Fletcher, 2014). In addition, only children may receive too much parental attention, and high levels of parental expectations and pressure to succeed. These will affect cognitive outcomes and non-cognitive outcomes such as psychological health and general well-being. For example, some evidence demonstrated that only children had slightly lower achievement (Huang and Wen, 2008), had lower subjective well-being (Fan, 2014; Wu, 2014) or had the same subjective well-being as non-only children (Huang J., 2012). Similarly, it has been consistently shown that there was no difference in academic self-efficacy between only children and non-only children (Huang W. L., 2012; Yuan, 2014).

In sum, only-child status may directly or indirectly influence child cognitive/non-cognitive development, and whether such an influence will be positive or negative haven't arrived at a consensus. Another important consideration is the selection of individuals into only-child families. Families that choose to have only one child may differ in parental characteristics. It is necessary to realize that only children are not a homogeneous category (Rosenberg and Hyde, 1993) and that social and economic factors (Falbo and Poston, 1993) must be taken into account when examining any data on only children.

### Moderating characteristics

The students' demographic characteristics (gender and region) and family characteristics (father's education level, mother's education level, family socio-economic status, parents' expectation, and family structure) may moderate the associations between only child status and cognitive/non-cognitive outcomes. It is very important to investigate the differences by demographic characteristics, because only children may have more strongly linked with cognitive/non-cognitive outcomes for certain subgroups of students. In addition, it is possible to compare students whose conditions are similar in many ways, but differ in only children conditions (e.g., the only children lived in an intact family compared with the non-only children in the same family).

There are a number of theoretical reasons to expect differences in the associations by demographic groups. First, gender and region may affect the associations between only child status and cognitive/non-cognitive outcomes. Because of the existence of gender preference, especially in rural area (e.g., Li and Lavely, 2003), different groups (girls and boys) may obtain different educational conditions, resulting in the differences between the two groups in the non-cognitive outcomes. For example, a study found that male only children in rural areas were associated with lower subjective well-being than male non-only children in the same areas, while there were no difference between girl only children and non-only children in rural areas (Wu, 2014). However, gender preference eliminated largely in one-child families, especially in urban China, the girls have the same educational conditions with boys (Tsui and Rich, 2002). In addition, one study found that only children in an urban area had higher academic performance than non-only children in the same type of area, while there was no difference in rural areas (Bao and Su, 1989; Poston and Falbo, 1990a,b).

Second, the characteristics of parents may affect the associations between only child status and cognitive/non-cognitive outcomes. Parental educational level may affected the parenting styles. The higher the parental educational level was, the more emotional warmth, understanding, and preference for their children they had (Lei et al., 2001; Li, 2001; Wang, 2005; Qiu and Han, 2010). Meanwhile, parenting styles were related to cognitive and non-cognitive outcomes. In particular, parental emotional warmth and understanding were positively related to academic performance, academic self-efficacy, and the well-being of children (Zhang et al., 2001; Hu et al., 2002; Wang and Ding, 2003; Yu and Yu, 2004; Chen et al., 2006; Yang, 2006; Zhu, 2012; Yan, 2014). Only children tended to receive more emotional warmth and understanding from their parents than non-only children (Zhang, 1998; Guo, 2013; Li, 2013). These findings suggest that parental educational level may affect the associations between only child status and cognitive/non-cognitive outcomes. In addition, parental educational expectations may affect the associations between only child status and cognitive/non-cognitive outcomes. Parents of only children usually had high expectations for their children (Man, 1993). Parental educational expectations could affect the children's educational behavior (Gong, 2004). When parental expectations for their children's education were higher, they would invest more energy (Zhan, 2006) and have a tendency to create a more supportive environment (Seginer, 1983).

Lastly, the characteristics of a family such as family socio-economic status and family structure may also affect the cognitive/non-cognitive outcomes between only children and non-only children. Higher-income families provided more educational resources and material safeguards such as living conditions, learning environment, course fees, etc. than lower-income families could (Coleman, 1988; Reed, 2012). In addition, only children in the intact/middle-income families had lower self-concept than non-only children with the same family, while there was no such difference in the broken/higher-income or lower-income families (Zeng, 2004).

### Current study

As an extension to former studies, this study systematically focused on the associations between only child status and their cognitive/non-cognitive outcomes and more importantly on these possible moderators, through a sample of junior middle school students. Analyses were conducted to consider whether gender, region, parental educational level, parental expectations, family socio-economic status, and family structure moderated the associations. These characteristics were chosen because they have been highlighted in previous research with relation to only child status or cognitive/non-cognitive outcomes. However, with the exception of gender and urban address, few studies have investigated whether these family characteristics moderate the associations. This study examined the heterogeneity within groups to investigate whether particular demographic characteristics moderated the associations more strongly than others. Through this approach, the present study may provide insights helping both academics and policy makers understand the associations between only child status and cognitive/non-cognitive outcomes.

## Methods

### Data and sample

Participants were students of seventh and eighth graders from 53 schools in a medium-sized city in the middle part of China (*N* = 6,240). They completed the Student questionnaire, which took about 40 min. Meanwhile, a member of his/her family (e.g., father, mother, grandfather, grandmother, sister, uncle, brother, and aunt, etc.) was required to complete the Parent questionnaire. All samples were randomly selected through stratified random sampling from a typical district with Chinese characteristics. First of all, a city with typical “Chinese characteristics” was picked. We divided the city into four groups and selected a district/county from each group randomly based on the GDP per capita, education funding, resident population, the proportion of urban population, and education performance. Then, 53 schools were chosen according to the attributes of each school (city, urban, rural), school funding resource (public and private), funding quantum, and the performance of students. Finally, 2–3 classes in Grades 7 and 8 were sorted out as determined by class category (balance class, special class), students' academic performance, sex ratio, etc.

Of these students and their family members, 488 were missing values with respect to outcome variables and were excluded from the analysis, resulting in a final sample of 5,752. The information on students, such as gender, grade, only child status, and boarding status was obtained from the Student questionnaire. Other information about the demographic characteristics of students, including registered permanent residence, parental educational level, parental expectations, family socio-economic status, and family structure, were from the Parent questionnaire.

### Measures

#### Cognitive outcomes

Given the strong relationship between academic performance and cognitive ability (Kuncel et al., 2004; Spinath et al., 2006; Richardson et al., 2012), we considered the performance of Chinese and mathematics in the second semester united standardized examinations as a proxy for cognitive outcomes. These covered Chinese basic knowledge and capability in reading and expression. The mathematics examination measured mathematical knowledge and problem-solving capability. The designation of tests was based on the school curriculum in China. For specific aspect, the united standardized examinations in three districts were different from another district's^1^. The scores in three districts ranged from 0 to 100 and those in another district were from 0 to 120. Higher scores reflected higher corresponding cognitive ability.

#### Non-cognitive outcomes

All items estimating non-cognitive outcomes were from the Student questionnaire.

##### School well-being (SWB)

The well-being questionnaire was divided into three subscales: Well-being at school, attitudes about school, and school belonging. The items for the well-being at school subscale were taken from the well-being questionnaire of Van Landeghem (1991), which had well-established validity and reliability. Items of other subscales were derived from the questionnaires of Program for International Student Assessment (PISA) (Organization for Economic Cooperation and Development [OECD], 2003). There were 4 items in the well-being at school subscales (e.g., “I like attending this school more than other schools”), 4 items in the attitudes about school subscale (e.g., “It is a waste of time to attend the school”), and 5 items in the school belonging subscales (e.g., “It is easy for me to make friends”). The scale had a range from 1 (very much disagree) to 4 (very much agree). Six items were reverse coded. In the present study, the scale reliability is 0.86.^2^

##### Academic self-efficacy (ASE)

Academic self-efficacy represents the belief that one can successfully learn and perform given academic tasks at designated levels (Schunk, 1991). It is assumed to influence the choice and pursuance of tasks and actions. To assess the extent of academic self-efficacy, we used the academic self-efficacy questionnaire with Cronbach's α between 0.752 and 0.829 (Liang, 2000). The questionnaire consisted of two parts: Learning ability and study behavior. Each part has 11 items. Self-efficacy of learning ability refers to students' assessment about whether they were competent in terms of task-related abilities and skills, and whether they believe they have a high probability of successful academic performance (e.g., “I believe I can get good results in study”). Self-efficacy of study behavior refers to the self-judgment of one's ability, namely they could take some learning methods to achieve goals (e.g., “I found I always let my mind wander that I can't listen carefully in class”). The scale of each item had a range from 1 (completely disagree with the description) to 4 (completely agree with the description). Higher scores signified a higher degree of academic self-efficacy. In the present study, the scale reliability is 0.87.

##### Academic self-concept (ASC)

Academic self-concept refers to the self-evaluation of one's general ability in a domain (Marsh and Martin, 2011). In this study, the students' ASC was measured by the academic self-concept questionnaire taken from the well-being questionnaire of Van Landeghem (1991), which has well-established validity and reliability. The questionnaire consists of 9 items with the same 4-point scale as above (alpha = 0.77). The response scale for each item ranged from 1 (completely disagree with the description) to 4 (completely agree with the description). There were five positive-coded items (e.g., “I think I am able to learn all courses of the school very well”) and four reverse-coded items (e.g., “I'm worried that I can't do well in the final exam”). Higher scores indicate a higher academic self-concept. The scale reliability in this investigation is 0.76. Although the reliability is relatively low, this questionnaire has been used in many studies (e.g., Opdenakker and Van Damme, 2000).

##### Academic motivation (AM)

Student motivation is considered to be a dynamic, multifaceted phenomenon (Graham and Weiner, 1996; Eccles et al., 1998; Seifert, 2004). The academic motivation questionnaire adopted in the current study was the one used by Li (2007). It is based on the social cognitive theory of motivation featuring three components of: “value,” “expectations,” and “emotional,” which all have well-established validity and reliability. The “value” belief is measured by three subscales: Intrinsic goal orientation (four items; e.g., “I prefer it if the content of this course is more challenging so I can learn new things.”), external goal orientation (four items; e.g., “I want to get better grades in this course than others if possible,”), the value of task (six items; e.g., “It is important to learn the contents of this course in the classroom for me.”). The expectations belief was measured by two subscales: Beliefs about learning control (four items; e.g., “As long as I learn appropriately, I can learn the content of the lesson.”), confidence about his/her ability and doing homework (eight items; e.g., “I'm sure I can get a good score on this course.”). Emotional state was measured by anxiety (five items; e.g., “I have an uneasy feeling in the exam.”). The scale had a range from 1 (very much disagree) to 4 (very much agree). The reliability of the scale in this investigation is good (α = 0.82).

#### Moderators

To consider whether student and family characteristics moderated the associations between only child status and cognitive/non-cognitive outcomes, analyses were stratified by students' gender, region, parental educational levels, parental expectations, family socio-economic status, and family structure. The students' gender was coded as girl and boy and their region was coded as “rural area” (agricultural registered permanent residence) and “urban area” (not agricultural registered permanent residence). Parental educational level was divided into mothers' educational level and fathers' educational level, and was coded as “lower-educated” (less than high school), “middle-educated” (high school), and “higher-educated” (college or more). Parental expectations concerning their children's education was coded as “lower-expected” (associate degree or less), “middle-expected” (university degree), and “higher-expected” (postgraduate degree)^3^. Family socio-economic status was coded as “lower-income” (annual income under 30,000 RMB), “middle-income” (annual income between 30,000 and 90,000 RMB), and “higher-income” (annual income more than 90,000 RMB). Family structure was coded as “intact family” (both father and mother) and “broken family” (either father or mother or another).

#### Covariates

This study also controlled for three covariates (grade, students' boarding status, and three-generation family households), which might affect the associations between only child status and their cognitive/non-cognitive outcomes. For example, a study found that the differences between only children and non-only children in cognitive ability gradually reduced with increasing grade (Ji et al., 1993). Similarly, only children were more doted on by their parents and grandparents in three-generation family households (Guan, 2000; Fong, 2004).

Students' grade was coded as grade 7 and grade 8. Students' boarding status was coded as “extern” and “boarder.” Co-residence was coded as “two-generation family households” and “three-generation family households” (the students lived with his or her own parent(s) and at least one grandparent (a grandmother, grandfather, or both).

### Analytic strategy

Before the data analysis, the moderators and covariates were transformed into dummy variables. Chi-square tests were used to compare the ratio between only children and non-only children in each moderating demographic group (see Table 1), while independent-sample *t*-tests were used to examine mean-level differences in the standardized cognitive and non-cognitive outcomes by only child status (see Tables 2-1, 2-2). Next, ordinary least squares regression models with extensive demographic controls were used to assess the associations between only child status and cognitive/non-cognitive outcomes. The model analyses were disaggregated by each moderating characteristic (students' gender and region, their parental education level, parental expectations, family socio-economic status, and family structure) and Chow tests were conducted to test whether the revealed associations were significantly different in each demographic characteristic (see Tables 3-1, 3-2).

**Table 1:** **Sample characteristics**.

**Variable**	**Full sample**	**Only children**	**Non-only children**
Only children (%)	32	—	—
**STUDENTS' DEMOGRAPHIC CHARACTERISTICS**			
Gender (%)			
Boys	53	*58*	*50*
Girls	47	*42*	*50*
Grade (%)			
Seven	53	51	54
Eight	47	49	46
Region (%)			
Urban	44	*79*	*28*
Rural	56	*21*	*72*
Boarding Condition (%)			
Home	60	*70*	*55*
School	40	*30*	*45*
Generation (%)			
Three	13	*17*	*12*
Two	87	*83*	*88*
**FAMILY CHARACTERISTICS**			
Father's education (%)			
Lower-educated	51	*23*	*64*
Middle-educated	24	24	24
Higher-educated	25	*53*	*12*
Mother's education (%)			
Lower-educated	58	*25*	*73*
Middle-educated	21	*26*	*18*
Higher-educated	22	*49*	*9*
Parents' expectation (%)			
Lower-expected	13	*7*	*16*
Middle-expected	33	*30*	*34*
Higher-expected	54	*63*	*50*
Socio-economic status (SES) (%)			
Lower-income	46	*27*	*55*
Middle-income	35	*46*	*30*
Higher-income	19	*27*	*15*
Family structure (%)			
Broken	20	22	20
Intact	80	78	80
*N*	5,752	1,827	3,925

*Chi-square tests of the proportion between only children and non-only children in each moderating demographic group were used. Figures in italics indicate statistically significant differences at p < 0.05*.

**Table 2-1:** **Means on cognitive outcomes by moderating characteristics and only children**.

	**Three districts**						**One district**					
			**Chinese**		**Mathematics**				**Chinese**		**Mathematics**	
**Variable**	***N***	**%O**	**O**	**NO**	**O**	**NO**	***N***	**%O**	**O**	**NO**	**O**	**NO**
Full sample	3,964	40	*0.22*	−*0.15*	*0.26*	−*0.17*	1,787	13	0.01	0.004	0.12	−0.01
**GENDER**												
Boys	2,079	45	0.03^*^	−0.46^*^	*0.21*	−*0.22*	946	15	−0.09^*^	−0.19^*^	0.09	−0.10
Girls	1,884	36	*0.51*	*0.14*	*0.33*	−*0.13*	841	11	0.18	0.21	0.16	0.08
**REGION**												
Rural	1,564	14	−0.39	−0.28	−0.36	−0.30	1,550	10	−0.08	−0.002	−0.001	−0.04
Urban	2,256	58	0.33^*^	0.05^*^	*0.36*	*0.03*	206	33	0.19^*^	−0.01^*^	0.41	0.25
**FATHER'S EDUCATION**												
Lower-educated	1,535	18	−0.26	−0.34	−0.38	−0.36	1,325	10	−0.07	−0.04	−0.07	−0.09
Middle-educated	1,017	37	0.01	−0.03	0.04	−0.07	352	16	0.07	0.13	0.34	0.22
Higher-educated	1,344	68	*0.47*	*0.24*	*0.56*	*0.25*	87	38	0.12	0.19	0.43	0.45
**MOTHER'S EDUCATION**												
Lower-educated	1,791	17	−0.32	−0.30	−0.33	−0.33	1,467	9	0.04	−0.02	0.01	−0.06
Middle-educated	944	43	0.07	0.01	0.10	0.02	218	22	−*0.16*	*0.18*	0.10	0.33
Higher-educated	1,180	73	*0.51*	*0.30*	*0.56*	*0.27*	68	52	0.30	0.35	0.62	0.42
**PARENTAL EXPECTATIONS**												
Lower-expected	418	21	−0.86	−0.95	−1.02	−0.94	298	11	−0.48	−0.44	−0.54	−0.67
Middle-expected	1,256	37	*0.03*	−*0.19*	−*0.03*	−*0.22*	573	11	−0.13	0.02	0.02	0.01
Higher-expected	2,197	46	*0.42*	*0.10*	*0.52*	*0.09*	881	15	0.22	0.16	0.35	0.20
**SOCIO-ECONOMIC STATUS**												
Lower-income	1,202	27	−0.22	−0.28	−0.27	−0.36	1,375	11	0.003	−0.01	0.05	−0.05
Middle-income	1,636	46	0.28^*^	−0.14^*^	*0.34*	−*0.09*	326	19	0.01^*^	0.09^*^	0.29	0.15
Higher-income	1,003	40	*0.46*	*0.08*	*0.50*	*0.06*	45	13	0.51	0.14	0.68	0.32
**FAMILY STRUCTURE**												
Broken	738	44	−*0.01*	−*0.20*	−0.13	−0.24	436	16	−0.28	−0.13	−0.14	−0.18
Intact	3,226	40	0.29^*^	−0.14^*^	*0.36*	−*0.16*	1,352	12	0.14^*^	0.05^*^	0.23	0.04

*O, only children; NO, non-only children. Tests of variances homogeneity were used to rule out groups with different variances. Statistically significant differences were noted with asterisks (p < 0.01). Independent-samples t-tests were used to examine mean-level differences in the standardized cognitive outcomes by only-child status. Statistically significant differences (p < 0.05 and Cohen's d > 0.20) are noted in italics*.

**Table 2-2:** **Means on non-cognitive outcomes by moderating characteristic and only children**.

			**Non-cognitive outcomes**									
			**SWB**		**ASE**		**ASC**		**AM1**		**AM2**	
**Variable**	***N***	**%O**	**O**	**NO**	**O**	**NO**	**O**	**NO**	**O**	**NO**	**O**	**NO**
Full sample	5,750	32	*0.18*	−*0.08*	*0.17*	−*0.08*	*0.17*	*0.08*	*0.12*	−*0.05*	−0.002	0.003
**GENDER**												
Boys	3,025	35	0.17^*^	−0.10^*^	0.27^*^	−0.004^*^	0.22^*^	−0.02^*^	0.14^*^	−0.04^*^	0.05	0.06
Girls	2,725	28	0.19^*^	−0.06^*^	*0.03*	−*0.16*	0.11^*^	−0.14^*^	*0.09*	−*0.07*	−0.08	−0.05
**REGION**												
Rural	3,114	12	−0.15	−0.14	−0.07	−0.14	−0.02	−0.10	−0.07	−0.09	−0.04	−0.01
Urban	2,462	56	*0.28*	*0.05*	0.22	0.04	0.22^*^	−0.05^*^	0.17	0.03	0.004	0.03
**FATHER'S EDUCATION**												
Lower-educated	2,860	14	−0.09	−0.16	−0.11	−0.13	−0.11	−0.1	−0.08	−0.09	−0.02	0.005
Middle-educated	1,369	32	0.13	0.008	0.11	−0.01	0.08	−0.03	0.08	−0.02	0.06	0.001
Higher-educated	1,431	66	*0.33*	*0.11*	*0.31*	*0.03*	*0.36*	*0.04*	*0.24*	*0.03*	−0.04	−0.03
**MOTHER'S EDUCATION**												
Lower-educated	3,258	13	−0.06	−0.14	−0.07	−0.12	−0.08	−0.12	−0.07	−0.08	0.004	0.005
Middle-educated	1,162	39	0.14	0.03	0.14	0.001	0.11	−0.01	0.13	0.003	0.04^*^	−0.002^*^
Higher-educated	1,248	72	0.34	0.14	*0.3*	*0.05*	*0.35*	*0.05*	*0.23*	*0.05*	−0.03	−0.02
**PARENTS' EXPECTATION**												
Lower-expected	716	17	−0.33	−0.32	−0.13	−0.32	−0.38	−0.37	−0.11	−0.23	0.09	−0.02
Middle-expected	1,829	29	0.11^*^	−0.12^*^	0.03^*^	−0.15^*^	−0.04	−0.18	0.03^*^	−0.09^*^	0.05	0.03
Higher-expected	3,078	37	0.29^*^	0.02^*^	0.26^*^	0.03^*^	0.35^*^	0.08^*^	0.20^*^	0.02^*^	−0.04	−0.01
**SOCIO-ECONOMIC STATUS**												
Lower-income	2,577	18	−0.09	−0.18	−0.03	−0.16	−0.08	−0.13	−0.05	−0.11	0.02	−0.01
Middle-income	1,962	41	0.3^*^	−0.005^*^	*0.18*	−*0.03*	0.24^*^	−0.04^*^	0.16^*^	−0.006^*^	−0.02^*^	0.006^*^
Higher-income	1,048	47	0.29	0.09	*0.33*	*0.07*	*0.34*	−*0.02*	*0.24*	*0.04*	0.001	0.02
**FAMILY STRUCTURE**												
Broken	1,174	33	0.01	−0.10	0.02	−0.13	−0.11	−0.15	−0.07	−0.10	0.007	0.03
Intact	4,578	31	0.24^*^	−0.06^*^	0.21^*^	−0.07^*^	0.24^*^	−0.06^*^	0.19^*^	−0.04^*^	−0.004^*^	−0.01^*^

*O, only children; NO, non-only children; SWB, school well-being; ASE, academic self-efficacy; ASC, academic self-concept; AM1, Internal academic motivation; AM2, External academic motivation. Tests of variances homogeneity were used to rule out groups with different variances. Statistically significant differences were noted with asterisks (p < 0.01). Independent-samples t-tests were used to examine mean-level differences in the standardized non-cognitive outcomes by only-child status. Statistically significant differences (p < 0.05 and Cohen's d > 0.20) were noted in italics*.

**Table 3-1:** **Only children on the cognitive outcomes from separate regressions stratified by gender, region, parental education, parental expectations, family socio-economic status, and family structure**.

	**Cognitive outcome (three districts)**		**Cognitive outcome (one district)**	
**Variable**	**Chinese**	**Mathematics**	**Chinese**	**Mathematics**
Full sample	0.04^*^ (0.03)	0.03^*^ (0.04)	−0.01 (0.02)	0.001 (0.03)
**GENDER**				
Boys	0.02 (0.05)	−0.01 (0.05)	−0.01 (0.03)	0.01 (0.03)
Girls	0.07^*^ (0.05)	0.10^***^ (0.05)	−0.01 (0.04)	−0.01 (0.04)
**REGION**				
Rural	[−0.03 (0.07)]	[−0.03 (0.07)]	−0.01 (0.03)	0.003 (0.03)
Urban	[0.07^***^ (0.04)]	[0.07^**^ (0.04)]	0.004 (0.05)	−0.04 (0.05)
**FATHER'S EDUCATION**				
Lower-educated	0.01 (0.07)	[−0.02 (0.07)]	0.01 (0.03)	0.003 (0.03)
Middle-educated	0.02 (0.07)	0.03 (0.07)	−0.06 (0.04)	−0.02 (0.04)
Higher-educated	0.06^*^ (0.05)	[0.08^**^ (0.05)]	−0.07 (0.07)	−0.07 (0.07)
**MOTHER'S EDUCATION**				
Lower-educated	[−0.01 (0.06)]	[−0.02 (0.07)]	[0.02 (0.03)]	0.01 (0.03)
Middle-educated	0.05 (0.06)	0.03 (0.06)	[−0.13^*^ (0.05)]	−0.01 (0.05)
Higher-educated	[0.07^*^ (0.05)]	[0.09^**^ (0.05)]	−0.08 (0.07)	0.31^*^ (0.06)
**PARENTAL EXPECTATIONS**				
Lower-expected	−0.03 (0.15)	[−0.07 (0.14)]	0.003 (0.07)	0.002 (0.08)
Middle-expected	0.06 (0.06)	0.01 (0.06)	−0.05 (0.05)	−0.05 (0.05)
Higher-expected	0.04 (0.04)	[0.07^**^ (0.04)]	0.01 (0.03)	0.02 (0.03)
**SOCIO-ECONOMIC STATUS**				
Lower-income	−0.01 (0.07)	0.01 (0.07)	0.02 (0.03)	0.01 (0.03)
Middle-income	0.05 (0.05)	0.03 (0.05)	−0.10 (0.05)	−0.04 (0.05)
Higher-income	0.06 (0.06)	0.06 (0.06)	0.21 (0.15)	0.01 (0.15)
**FAMILY STRUCTURE**				
Broken	0.01 (0.08)	[−0.08 (0.08)]	−0.05 (0.06)	−0.01 (0.05)
Intact	0.04^*^ (0.04)	[0.07^**^ (0.04)]	0.01 (0.03)	0.004 (0.03)

Values are beta coefficients of only child status on cognitive outcomes in a series of regression models. Standard errors (SE) are in parentheses. All regressions control for grade, region, three-generation family, boarding condition, gender, parental education, parental expectations, socio-economic status, and family structure. Results of three districts from Chow tests found the following significant differences (p < 0.05, in the square brackets): urban vs. rural for Chinese and mathematics; about father's educational level, higher- vs. lower- educated for mathematics, about mother's education level, higher- vs. lower- educated for Chinese and mathematics; about parental expectations, higher- vs. low-ranking for mathematics, higher- vs. middle-ranking for mathematics; about family structure, broken vs. intact for mathematics. Results of one district: about mother's education level, middle- vs. lower- educated for Chinese.

* p < 0.05.

** p < 0.01.

*** *p < 0.001*.

**Table 3-2:** **Only children on the non-cognitive outcomes from separate regressions stratified by gender, region, parental education, parental expectations, family socio-economic status, and family structure**.

		**Non-cognitive outcome**			
**Variable**	**SWB**	**ASE**	**ASC**	**AM1**	**AM2**
Full sample	0.04^*^ (0.03)	0.03^*^ (0.03)	0.06^***^ (0.03)	0.03^*^ (0.03)	−0.02 (0.03)
**GENDER**					
Boys	0.01 (0.05)	0.01 (0.05)	0.02 (0.05)	0.004 (0.04)	−0.01 (0.04)
Girls	0.07^**^ (0.05)	0.06^*^ (0.05)	0.10^***^ (0.05)	0.07^**^ (0.04)	−0.03 (0.04)
**REGION**					
Rural	[−0.01 (0.05)]	0.01 (0.05)	0.01 (0.05)	−0.009 (0.04)	−0.03 (0.04)
Urban	[0.07^**^ (0.05)]	0.04 (0.05)	0.08^***^ (0.05)	0.06^**^ (0.04)	−0.004 (0.03)
**FATHER'S EDUCATION**					
Lower-educated	0.02 (0.05)	[−0.01 (0.05)]	[0.01 (0.05)]	[−0.02 (0.04)]	−0.04 (0.04)
Middle-educated	0.02 (0.06)	[0.01 (0.06)]	[0.03 (0.06)]	0.02 (0.05)	0.01 (0.05)
Higher-educated	0.05 (0.07)	[0.08^**^ (0.07)]	[0.1^***^ (0.06)]	[0.09^**^ (0.05)]	−0.004 (0.05)
**MOTHER'S EDUCATION**					
Lower-educated	0.01 (0.05)	[−0.02 (0.05)]	[0.007 (0.05)]	[−0.03 (0.04)]	−0.03 (0.04)
Middle-educated	0.05 (0.06)	0.06^*^ (0.06)	0.06^***^ (0.06)	[0.08^*^ (0.05)]	0.02 (0.05)
Higher-educated	0.05 (0.07)	[0.07^*^ (0.07)]	[0.09^**^ (0.07)]	[0.07^*^ (0.06)]	−0.02 (0.05)
**PARENTAL EXPECTATIONS**					
Lower-expected	−0.009 (0.09)	0.05 (0.1)	−0.03 (0.09)	0.02 (0.08)	0.04 (0.08)
Middle-expected	0.07^*^ (0.06)	0.04 (0.06)	0.08^**^ (0.06)	0.05 (0.04)	−0.02 (0.04)
Higher-expected	0.03 (0.05)	0.03 (0.05)	0.06^*^ (0.05)	0.03 (0.04)	−0.03 (0.04)
**SOCIO-ECONOMIC STATUS**					
Lower-income	[−0.01 (0.05)]	0.01 (0.05)	[−0.004 (0.05)]	−0.008 (0.04)	−0.002 (0.04)
Middle-income	[0.09^*^ (0.06)]	0.03 (0.06)	[0.09^*^ (0.05)]	0.05 (0.04)	−0.03 (0.04)
Higher-income	0.03 (0.08)	0.06 (0.08)	[0.09^*^ (0.08)]	0.07 (0.07)	−0.01 (0.06)
**FAMILY STRUCTURE**					
Broken	0.03 (0.08)	0.000 (0.08)	[−0.03 (0.08)]	[−0.04 (0.06)]	−0.05 (0.06)
Intact	0.05^*^ (0.04)	0.04^*^ (0.04)	[0.08^***^ (0.04)]	[0.06^**^ (0.03)]	−0.004 (0.03)

Values are beta coefficients of only child status on non-cognitive outcomes in a series of regression models. Standard errors (SE) are in parentheses. All regressions control for grade, region, three-generation family, boarding condition, gender, parents' education, parents' expectation, socio-economic status, and family structure. Results from Chow tests found the following significant differences (p < 0.05, in the square brackets): urban vs. rural for school well-being; about father's educational level, higher vs. middle- and lower- educated for academic self-efficacy and academic self-concept; higher- vs. lower- educated for internal academic motivation; about mother's education level, higher- vs. lower- educated for academic self-efficacy, academic self-concept and internal academic motivation; middle vs. lower-educated for internal academic motivation; about socio-economic status (SES), middle vs. low-status for school well-being, academic self-concept; higher vs. low-status for academic self-concept; about family structure, broken vs. intact family for academic self-concept and internal academic motivation.

* p < 0.05.

** p < 0.01.

*** p < 0.001

## Results

### Descriptive statistics on students and their families

Table 1 presents descriptive statistics, including differences by demographic characteristics for the full sample, only children, and non-only children. The results of a series of Chi-square tests between only child status and each moderating demographic group show that only children are more likely to be boys, in an urban area, living at home and have higher educated fathers. They are also more likely to have mothers with higher or middle education, parents' expectation of a postgraduate degree, be in families with higher or middle-income.

Table 2-1 shows the percentage of only children in each moderating demographic group and mean differences in the standardized cognitive outcomes by only child status for each of the moderating demographic groups. Results in the three districts showed that there was a significant difference between only children and non-only children in Chinese and mathematics. We found that female only children were significantly better at Chinese and mathematics. The results were the same for the male group. In urban areas, we also found the same results. Only children with a higher-educated father had significantly higher Chinese and mathematics scores than those of non-only children. The results were the same for the group with a higher-educated mother. We also found the same results for the group living in middle-income and higher-income families. In addition, the results were the same for the group with middle-ranking parental expectations and higher-ranking parental expectations. Only children lived in intact families had better Chinese and mathematics than non-only children of the same status. We only found that only children who lived in broken families had better Chinese than non-only children of the same status. However, results in one district showed that only children with a middle-educated mother had significantly higher Chinese scores than those of non-only children^4^.

Table 2-2 shows the percentage of only children in each moderating demographic group and mean differences in the standardized non-cognitive outcomes by only child status for each of the moderating demographic groups. For the full sample, only children had significantly higher school well-being, academic self-efficacy, academic self-concept, and internal academic motivation, as compared with non-only children. When stratified by gender, we found that female only children had significantly higher school well-being, academic self-efficacy, academic self-concept, and internal academic motivation than non-only children. The results were the same for the male group. In urban areas, the results showed that only children had significantly higher school well-being and academic self-concept compared with non-only children. Only children with a higher-educated father had significantly higher school well-being, academic self-concept, academic self-efficacy, and internal academic motivation than those of non-only children. When the mothers' educational level was categorized as higher educated, only children had significantly better academic self-concept, academic self-efficacy, and internal academic motivation. Only children who lived in middle-income families had higher school well-being, academic self-concept, academic self-efficacy, and internal academic motivation than non-only children. Only children living in higher-income families were significantly better in academic self-concept, academic self-efficacy, and internal academic motivation than non-only children of the same status. When stratified by parental expectation, only children were significantly better in school well-being, academic self-concept, academic self-efficacy, and internal academic motivation than non-only children when their parents' expectation was higher. Only children were significantly better in school well-being than non-only children with middle-ranking parental expectations. Lastly, the scores of only children who came from an intact family scored significantly better in school well-being, academic self-concept, academic self-efficacy, and internal academic motivation than non-only children who were from the same type of family.

### Associations between only child status and cognitive/non-cognitive outcomes

Table 3-1 shows the coefficients of only-child status in regression analyses on the cognitive outcomes for the full sample and sub-samples after stratification by our demographic characteristics of interest. In the results of three districts, the comparison of coefficients between urban vs. rural area showed that the associations for the urban students was significantly different from the rural students. Only children in an urban area were associated with higher Chinese and mathematics scores than non-only children in the same area. In rural areas, there was no difference between only child status and cognitive outcomes (Chinese and mathematics).

A similar analysis found that the association for students with higher-educated mothers was significantly different from those with lower-educated mothers. Only children with higher-educated mothers had higher Chinese and mathematics scores than non-only children with the same mothers. However, there was no difference between only child status and cognitive outcomes (Chinese and mathematics) in students with lower educated mothers. For the mathematics, the comparison of coefficients among the students who have the higher vs. middle vs. lower-educated fathers demonstrated that the association for the students with higher-educated fathers was significantly different from those with lower-educated fathers. Only children with higher-educated fathers were associated with higher mathematics scores than non-only children with the same fathers. While for the students with lower-educated fathers, there was no difference between only child status and mathematics.

We also found the same results for students with higher-ranking parental expectations compared with lower-ranking parental expectations and for the students in intact families compared with broken families. Only children had higher scores in mathematics than non-only children with high-ranking parental expectations. However, with lower-ranking parental expectations, there was no difference between only child status and mathematics. Only children in an intact family had higher mathematics scores than non-only children in the same family. Yet, in broken families, there was no difference between only child status and mathematics.

In one district results, a similar analysis found that the association for students with middle-educated mothers was significantly different from those with lower-educated mothers. Only children with middle-educated mothers had lower performance in Chinese than non-only children with the same mothers. However, there was no difference between only child status and Chinese^5^ in students with lower-educated mothers.

Table 3-2 shows the coefficients of only-child status in regression analyses on the non-cognitive outcomes for the full sample and sub-samples after stratification by our demographic characteristics of interest. For school well-being, the comparison of coefficients between urban vs. rural area showed that the associations for the urban students was significantly different from the rural students. Only children in an urban area were associated with higher school well-being than non-only children in the same area. In rural areas, there was no difference between only child status and school well-being. A similar analysis found that the association for students in middle-income families was significantly different from those with lower-income families. Only children in middle-income families had higher school well-being than non-only children in the same family type. However, in lower-income families, there was no difference between only child status and school well-being.

For academic self-efficacy and academic self-concept, a similar comparison showed that the association for the students with higher-educated fathers was significantly different from those with middle or lower-educated fathers. Meanwhile, the association for the students with higher-educated mothers was significantly different from those with lower-educated mothers. Only children with higher-educated fathers or mothers were associated with higher academic self-efficacy and academic self-concept than non-only children with the same type of parents. However, only children with middle or lower-educated fathers scored the same as non-only children with the same type of fathers in academic self-efficacy and academic self-concept. Only children with lower-educated mothers scored the same as non-only children with the same type of mothers in academic self-efficacy and academic self-concept. Only for academic self-concept, did we find that the association for the students from the higher or middle-income families was significantly different from those from lower-income families. Only children in higher or middle-income families had higher academic self-concept as compared with non-only children in the same family type. While in the lower-income families, there was no difference between only child status and academic self-concept. In addition, the association for the students in the intact families was significantly different from those in the broken families. Only children in the intact families had higher academic self-concept as compared with non-only children in the same families. For those in the broken families, there was no difference between only-child status and academic self-concept.

For internal academic motivation, the association for the students with higher-educated fathers was significantly different from those with lower-educated fathers. Only children with higher-educated fathers had higher internal academic motivation as compared with non-only children with fathers educated to the same level. For those with lower-educated fathers, there was no difference between only-child status and internal academic motivation. The association for the students with higher or middle-educated mothers was significantly different from those with lower-educated mothers. Only children with higher or middle-educated mothers had higher internal academic motivation as compared with non-only children with mothers educated to the same level. For those with lower-educated mothers, there was no difference between only child status and internal academic motivation.

The association for the students in the intact families was significantly different from those in the broken families. Only children in the intact families had higher internal academic motivation as compared with non-only children in the same families. For those in the broken families, there was no difference between only-child status and internal academic motivation.

## Discussion

This study has examined the associations between only child status and cognitive/non-cognitive outcomes using a large representative sample. We also introduced several moderating characteristics on these associations and thus it was possible to understand differences within parameters such as lower-ranking parental expectations vs. higher-ranking parental expectations and demographic groups. To our knowledge, it is the first study to investigate heterogeneity in these associations by parental educational level, parental expectations, family socio-economic status and family structure.

For cognitive outcomes, only children had better academic performance in Chinese and mathematics compared with non-only children in the sample of three districts' students. Analyses that studied differences by demographic groups found that region, parental education, parental expectations and family structure moderated the associations between only child status and cognitive outcomes, while gender and family socio-economic status did not. For non-cognitive outcomes, only children had higher school well-being, academic self-efficacy, academic self-concept, and internal academic motivation as compared with non-only children in the full sample of students. Region, parental education, family socio-economic status, and family structure moderated the associations between only child status and non-cognitive outcomes, while gender and parental expectations did not.

### Only child status in china

For the full sample of students, the results showed that only children were more likely to be boys as compared with non-only children. We tentatively suggest that the different degrees of son preference in urban and rural China drive this difference. The phenomenon that only children are more likely to exist in an urban area is consistent with the effects of the one-child policy. According to the policy, a second birth is approved and permitted by the government under certain conditions, such as when urban families have disabled firstborns or are blended families in which one parent has no biological offspring. In rural areas, some families can have another child, depending on their location, firstborn's sex, parental professions, and duration after the birth of the firstborn (White and White, 2012). In addition, life and attitudes, as well as the degree of policy implementation, were different in the urban areas of China than in the rural areas. With the development of society, more and more urban dwellers realize that one child is sufficient, because limiting family size allows for a higher standard of living and greater family happiness.

The results showed that only children were more likely to have fathers are higher-educated and mothers are higher or middle-educated. This phenomenon may be explained in two ways. On the one hand, parents with higher education are more likely to accept the only-child policy. On the other hand, most of the parents in our study were born in the 70's, when the state was the only source of work after graduating from college. This made it possible for the government to use various methods to promote the only-child policy, such as social pressure and employment limitations which were placed on parents (Kane and Choi, 1999).

The results showed that only children were more likely to occur in the group whose parental expectations was a postgraduate degree. This phenomenon conforms to the Confucian tradition which stresses the importance of education, such child-centered values were encouraged by an increasingly urgent sense that the family is the most reliable “welfare agency” for its members. Because the single child will be the only person who is responsible for supporting and caring for his/her parents (in some cases grandparents), his or her academic and career success has become the major concern of the family (Ming and Rich, 2002). Similarly, some of the respondents described feeling pressure to succeed because they were the only child in their families and were thus the sole recipient of parental expectations (Roberts and Blanton, 2001).

### The association between only child status and cognitive/non-cognitive outcomes

For cognitive outcomes, the results showed that being an only child was associated with higher levels of academic performance in Chinese and mathematics in the sample of three districts' students. This is consistent with reports made by others using data from China as well as other countries (e.g., Poston and Falbo, 1990a,b; Falbo and Poston, 1993), while not being in line with a recent study (Liu et al., 2010). For non-cognitive outcomes, the results showed that being an only child was associated with higher level of school well-being, academic self-efficacy, academic self-concept, and internal academic motivation as compared with non-only children, which is consistent with previous results (Hu, 2007, 2009; Kwan and Ip, 2009; Shao et al., 2013). However, the result is inconsistent with previous findings that being an only child was associated with lower subjective well-being (Wu, 2014) and does not support “Little Emperors” story (Fong, 2004).

These results can be explained in two ways: On the one hand, only children receive more attention from their parents (Schachter, 1959). Higher levels of parental attention or responsiveness help children develop a sense of security, which facilitates their development of intellectual competence, psychological confidence and mature behavioral patterns (Falbo, 1987). Attachment bonds with parents during adolescence continue to provide a solid basis for adolescents' psychological well-being (Ainsworth, 1982; Armsden and Greenberg, 1987). On the other hand, without the competition with siblings, their status as the only recipient of family assets is also conducive to their educational opportunities, physical health, as well as their general well-being (Falbo and Polit, 1986; Falbo, 1987; Liu et al., 2010). The results suggest that only children have more positive outcomes than non-only children both in cognitive and non-cognitive outcomes.

### The heterogeneity in association between only child status and cognitive outcomes

#### Region

The results found that region moderated the associations between only child status and cognitive outcomes. In the urban areas, only children were associated with better Chinese and mathematics than non-only children and the association was significantly different from that found in rural areas. However, we didn't find the superiority of only children in their academic performance in rural areas. The result is consistent with previous findings (Bao and Su, 1989; Poston and Falbo, 1990a,b). It also means that the advantages of only children in their academic performance were also affected by other factors, such as educational resources and ideology (Coleman, 1988; Zhang, 1997; Huang and Wen, 2008). Specifically, the education resources in rural area are limited and the education concept in rural area is relatively backward. Accordingly, the impact on only children is the same as on non-only children. These results may suggest that the advantage of only children in urban area on the cognitive outcomes is more obvious than that in rural area.

#### Parental educational level

These results showed that the parental educational level moderated the associations between only children and cognitive outcomes. Only children with higher-educated mothers were associated with improved Chinese and mathematics and the association was significantly different from those with lower-educated mothers. Meanwhile, in mathematics we found the same results by the father's educational level. These may suggest that the parental educational level has more effect on the students' academic achievement, especially for only children. The results may be explained that only children may receive more time and energy from their parents than non-only children, especially mothers (Chen, 1986; Feng, 1993). In addition, children tend to have a more intimate emotional relationship with their mother, and a more strict and formal relationship with their father (Pipp et al., 1985). If the parental educational level is higher, the effect of counseling may be better.

#### Parental expectations

The results found that parental expectations moderated the association between only child status and mathematics. Only children with higher status expectations had higher mathematics scores. In addition, the association was significantly different from those with lower-status and middle-status expectations. The results meant that parental educational expectations could affect the children's educational behavior (Gong, 2004). When parental expectations for their children's education were higher, they invested more energy (Zhan, 2006) and have a tendency to create a more supportive environment for their children (Seginer, 1983). In addition, only children as the only recipient of family assets could receive more resources from their parents than the children with siblings.

#### Family structure

The results found that family structure moderated the associations between only child status and mathematics. In the intact families, only children were associated with better mathematics than non-only children and the association was significantly different from that found in the broken families. The result revealed that the integrity of family structure could help only children achieve higher mathematics. The superiority of the only children didn't exist in the broken families. This phenomenon may be explained that parents or primary caregivers have no energy to care for their children's education. While in the intact families, only children receive more attention and family assets from their parents, which is also conducive to their educational opportunities (Schachter, 1959; Falbo and Polit, 1986; Falbo, 1987; Liu et al., 2010).

### The heterogeneity in association between only child status and non-cognitive outcomes

#### Region

Region moderated the associations between only child status and school well-being. In the urban areas, only children were associated with better school well-being than non-only children and the association was significantly different from that found in rural areas. The underlying explanation for this pattern may be different in preference for sons in rural China (e.g., Li and Lavely, 2003). This is because in traditional Chinese families, male offspring were favored over females as they were able to not only help with their parents work but also carry on the family name. An only son in a rural family is more likely to be doted on and have high expectations. Although an only son in a rural family would receive more resources and attention from their parents, they are under the huge pressure to succeed. Hence, for school well-being there was no significant difference between only children and non-only children in rural China. In urban China, where the son preference is not as prevalent, the gender disparity is not apparent. Meanwhile, only children, as the sole recipients, receive more resources and attention from their parents.

#### Parental educational level

For those students with higher-educated parents, only children were associated with more academic self-efficacy, academic self-concept and internal academic motivation. In addition, the association was significantly different from those with lower-educated parents. Together, these analyses suggest that the higher the parental educational level is, the greater differences are between only children and non-only children in academic self-efficacy, academic self-concept, and internal academic motivation. Possibly, these results can be indirectly supported by some previous studies. For example, Li (2004) found that there was a positive correlation between parent-child communication and internal academic motivation, while Falbo (2012) found that only children had better relations with their parents.

#### Family socio-economic status

The results showed that only children in middle-income families were associated with more school well-being and academic self-concept. In addition, the association was significantly different from those in lower-income families. These may suggest that the superiority of only children exist in middle-income families. These results indirectly support the resource dilution theory. Because only children didn't compete with siblings for scarce economic, they reported better adjustments both psychologically and behaviorally than non-only children (Liu et al., 2010). It is worth noting that there was little difference between only child status and school well-being, when the resources were abundant or scarce. The result was consistent with previous findings that the improvement of income level was not the sufficient condition to increase everyone's well-being (Easterlin, 2001; Diener et al., 2010). A recent study found that the influence of family socio-economic status on the adolescents' subjective well-being was affected by family education support and parent-child relationship (Zhang, 2016). This issue may be worth further study.

#### Family structure

The results found that family structure moderated the associations between only child status and academic self-concept. Only children in the intact families were associated with higher academic self-concept. In addition, the association was significantly different from those in broken families. For the internal academic motivation, we found the same results. These results revealed that the integrity of family structure could help only children have higher academic self-concept and internal academic motivation. In the intact families, only children receive more attention and family assets from their parents, which is also conducive to their educational opportunities, physical health (Schachter, 1959; Falbo and Polit, 1986; Falbo, 1987; Liu et al., 2010). However, in the broken families, children got more refusals and denials from their parents. Meanwhile, they received less emotional warmth and understanding. Parental emotional warmth and understanding were related to self-concept (Lei et al., 2001). In addition, the result is very interesting that there is no difference on academic self-efficacy. This issue might be further examined in future studies.

### Educational implications from the current investigation

With the implementation of the one-child policy for more than 30 years in China, it is important to deal with the education of only children. On the basis of research findings, several implications can be made for clinical work with only children and their parents. First, given the empirical basis for people's negative perceptions of only children, one of the most significant implications for those who work with only children or their parents is to educate them on the inaccuracies of the stereotypes about only children and provide correct information about the strengths and weaknesses of only children. Second, it is necessary to input more education resources to the rural area and take some effective measures to improve the rural people's education concept. For example, some courses about family education should be developed in primary and secondary schools. Parents should get the skills to apply a more scientific and objective perspective toward the education of only children, especially in rural areas. Third, it is necessary to take effective measures to improve the overall level of parents. The future of China's families will need to include more formal support for broad-based education, health, and continued emphasis on the opening of opportunities in rural and urban communities. Furthermore, in broken families, parents should not ignore their children's living condition and they should give children more warmth and understanding, which may promote their learning and growth. However, it may be that some parents pay too much attention to their children's cognitive and non-cognitive outcomes, especially in academic performance, academic self-concept, and internal academic motivation. Finally, parents should be aware of the tendency that they may place high expectations on their children. They should evaluate whether their expectations are realistic. If parental expectations are too high, it can lead to extreme stress and difficulties coping.

### Limitations

This study has a number of limitations. First, the sample was taken from the schools of a provincial city in the middle part of China. It is possible that these results are limited to this area so care should be taken when generalizing them at other locations. Moreover, the data are based on self-reporting, which may have the effect of artificially inflating associations among the variables. Given the cross-sectional character of our study, conclusions allow only associations and not causations. Finally, this research discussed some moderators between only child status and cognitive/non-cognitive outcomes. It is possible, that other variables, which we did not consider, influenced the associations. In addition, we didn't take into account the effect of birth order in the non-only children and compared only children with first born children and, separately, with children who were born as second. Future studies should build on these findings using longitudinal information that allow for a time lag between moderator and outcome variables.

## Conclusion

Despite these limitations, our study is important as it adds to the literature in the following ways. The findings from this study suggest that, in general, the associations between only child status and cognitive outcomes are moderated by region, parental education, parental expectations, and family structure, while gender and socio-economic status did not. Meanwhile, the associations between only child status and non-cognitive outcomes are moderated by region, parental education, socio-economic status, and family structure. This research shows that gender may be less signifcant moderator of the association between only child status and cognitive/non-cognitive outcomes despite large differences in the prevalence of only child status by these demographic characteristics.

This study also leads to more avenues for further research. It is important to continue studying only children beyond adolescence and examine how they perform in their adult roles in career and family. In addition, it would be interesting to examine the associations between the second generation of only child status and cognitive and non-cognitive outcomes. Furthermore, with the increase in the number of only children in rural areas, more research needs to be done to understand the situation of only children there.

## Ethics statement

The study was consistent with the ethical principles of human subjects. First, we told the detailed content of the study to the participants. Second, participants signed the informed consent on a voluntary basis. For minors, we got the consent of their parents before doing the research.

## Author contributions

NL was involved in study design, data analysis, and manuscript writing. YC was involved in study design, data acquisition, and analysis. XY and YH were involved in study design and commented on the manuscript.

### Conflict of interest statement

The authors declare that the research was conducted in the absence of any commercial or financial relationships that could be construed as a potential conflict of interest.

## Footnotes

^1^In results, analysis on cognitive outcomes is carried out respectively because of the inconsistent standardized examinations.

^2^The subscales of school well-being were selected from existing research scales, which had well-established validity and reliability. Hence, this research used it directly. Another published article also follows this approach (e.g., Pilkauskas, 2014).

^3^Parental expectations regarding their children's education do not match those parents' educational level. This situation is consistent with China's reality. In this study, most of the students were born in 2000–2001 and their parents were born in mid-1970s. With the development of society, people's education level increasingly upgrades. Therefore, the distribution of the parents' educational level is different from that of children's.

^4^Owing to the limited sample size in one district, the reliability of the results should be further confirmed.

^5^Owing to the limited sample size in one district, the reliability of the results should be further confirmed.
